# Synthesis and Cytotoxicity Studies of Poly(1,4-butanediol citrate) Gels for Cell Culturing

**DOI:** 10.3390/gels9080628

**Published:** 2023-08-04

**Authors:** Aleksandra Bandzerewicz, Klara Niebuda, Agnieszka Gadomska-Gajadhur

**Affiliations:** Faculty of Chemistry, Warsaw University of Technology, 00-662 Warsaw, Poland; aleksandra.bandzerewicz.dokt@pw.edu.pl (A.B.); klara.niebuda.stud@pw.edu.pl (K.N.)

**Keywords:** poly(1,4-butanediol citrate), biomaterials, polyesters, optimisation, polymer films, cytotoxicity

## Abstract

One of the main branches of regenerative medicine is biomaterials research, which is designed to develop and study materials for regenerative therapies, controlled drug delivery systems, wound dressings, etc. Research is continually being conducted to find biomaterials—especially polymers—with better biocompatibility, broader modification possibilities and better application properties. This study describes a potential biomaterial, poly(1,4-butanediol citrate). The gelation time of poly(1,4-butanediol citrate) was estimated. Based on this, the limiting reaction time and temperature were determined to avoid gelling of the reaction mixture. Experiments with different process conditions were carried out, and the products were characterised through NMR spectra analysis. Using statistical methods, the functions were defined, describing the dependence of the degree of esterification of the acid groups on the following process parameters: temperature and COOH/OH group ratio. Polymer films from the synthesised polyester were prepared and characterised. The main focus was assessing the initial biocompatibility of the materials.

## 1. Introduction

Biomaterials constitute a specific group of materials that vary in their chemical composition and properties, but have one main feature in common: safety for use in the human body. It allows them to serve as fillers or substitutes for tissues and organs. There are three main groups of biomaterials: ceramic, metallic and polymeric [1,2,3,4,5].

In tissue engineering, biomaterials are used to produce cell scaffolds, an artificial replacement of the extracellular matrix in the regenerating tissue. The choice of material depends on the type of tissue to be regenerated. Ceramic materials are used for bone tissue reconstruction as they have good biocompatibility [3,4,6,7]. The applicability of bioceramics in tissue engineering is nonetheless narrowed as there are difficulties in shaping them for implantation due to their non-flexibility, rigidity, as well as the hardness and brittleness of the surface [6]. Metallic materials are used primarily in dentistry and orthopaedics, mainly titanium, chromium, cobalt and magnesium or their alloys. Cellular scaffolds made of titanium are mechanically strong and resistant to prolonged stress and corrosion. A limitation of using metallic materials is the possibility of an allergic reaction [8,9,10]. Polymers are the most common materials for cellular scaffolds [4,6,11,12,13,14,15,16].

Polymeric materials have found extensive applications in tissue engineering due to the wide variety of physio-chemical and mechanical modification possibilities. In order to be used for medical purposes, they must meet specific criteria [6]. In the case of biodegradable polymers, aside from them being biocompatible, it is necessary that their degradation products are non-toxic and, in addition, bioresorbable—i.e., metabolisable by the organism. Additionally, the degradation time of the material should be as close as possible to that of the regeneration process. The mechanical properties of the degradable material should change according to the course of the tissue reconstruction process. Materials for external applications should be insensitive to cleaning and antiseptic substances. Polymers will have different physical and mechanical property requirements depending on the type of tissue to be regenerated [11,17,18,19,20,21,22].

One of the limitations of using natural polymers is the risk of contamination by microorganisms, such as fungi or bacteria. In addition, due to their zoonotic origin, they can cause an allergic reaction in humans [17]. On the contrary, synthetic polymers have a consistent composition and are easier to process into scaffolds. An important group of materials is polyesters; among which, poly(diols citrates) have recently gained importance [23,24,25,26,27,28,29].

Citric acid is a readily available, cheap and non-toxic compound that plays an essential role in the metabolism of cells in living organisms—its ionised form is an intermediate in the Krebs cycle. The multi-functionality of the citric acid molecule (i.e., three carboxyl and one hydroxyl group) is an important asset. Firstly, it can react with diol monomers in a polycondensation reaction. The synthesis does not require a catalyst. Secondly, unreacted side groups in the prepolymer can later be used for crosslinking the polymer chain or for surface modification of the material [29]. An important aspect of the design of citrate-based biomaterials is the choice of dihydroxyl alcohol, which affects the product’s properties. Studies have shown that in the group of diols containing 4–12 carbon atoms in the aliphatic chain, the longer the aliphatic chain increases, the more flexible the material. In the case of diols with 12–16 carbon atoms in the chain, it is possible to obtain a material exhibiting shape memory [27,29,30].

The majority of research to date has focused on poly(1,8-octanediol citrate) (POC). POC-based materials show hemocompatibility and reduce the risk of thrombus formation compared to the commonly used polymers [24,29,31,32,33]. This work covers the synthesis of poly(1,4-butanediol citrate), its mathematical description and the preparation and testing of polymer films. After further research and functional modifications, such films could be used, for example, as dressings or cell culture media.

## 2. Results and Discussion

**Figure 1:**
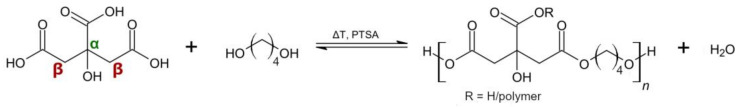
The synthesis of poly(1,4-butanediol citrate); α-C(O)O-H and β-C(O)O-H groups are marked.

The main objective of this study was to carry out the synthesis of poly(1,4-butanediol citrate) from citric acid and 1,4-butanediol in the presence of a PTSA catalyst (Figure 1) and to determine its optimum conditions at varying temperatures and COOH/OH ratios. The output variable is the degree of esterification of the carboxyl groups of citric acid. Due to differences in the reactivity of the acid groups resulting from their position in the molecule, steric hindrance and the different ordering of the carbon atoms, they were referred to as α-C(O)O-H and β-C(O)O-H groups, as shown in Figure 1.

**Figure 2:**
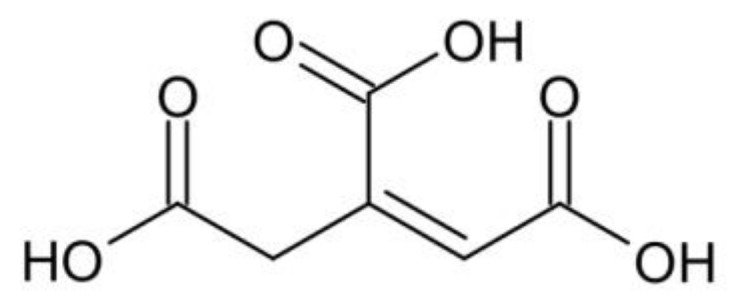
Aconitic acid.

In the structure of citric acid, there is one hydroxyl group, in addition to the three carboxyl groups. For this reason, the homopolycondensation or dehydration of citric acid to aconitic acid is possible (Figure 2). In the studies described herein, the conversion of the OH group is assumed to be negligible. Its influence is neglected in all calculations due to its location and high steric hindrance. This group is more difficult to access than the OH groups in the diol, which will be more easily esterified.

The conditions under which the maximum degree of esterification of the carboxyl groups is obtained without gelling the reaction mixture were considered the optimal point of synthesis. Based on preliminary studies, the temperature and time limits for the synthesis of poly(1,4-butanediol citrate) from citric acid and 1,4-butanediol were determined. A fixed reaction running time of 50 min was established. Reactions were carried out in the presence of a PTSA catalyst, the addition of which was constant and always counted as 1% of the weight of the citric acid.

### 2.1. Spectral Analysis of the Products

**Figure 3:**
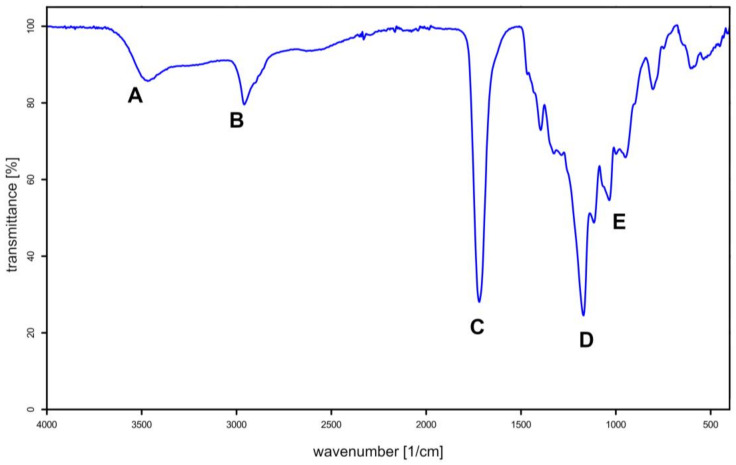
FTIR spectrum of selected poly(1,4-butanediol citrate) sample.

The infrared spectroscopy spectrum of the chosen synthesis product was measured (COOH/OH molar ratio was 1.0). Characteristic bands were marked to confirm the successful formation of polyester (Figure 3).

The obtained spectrum shows:

- a mid-intensity broad band around 3500 cm^−1^ (**A**) originating from an O-H bond in the terminal groups of the oligomer chains (diol fragments) and the free hydroxyl group in the acid; the broadening of the band occurs due to the presence of hydrogen bonds;
- a band at about 2950 cm^−1^ (**B**) due to the stretching vibrations in C-H bonds of the aliphatic groups;
- a narrow band of high intensity near 1715 cm^−1^ (**C**) originating from the carbonyl group in ester; the right-side-broadening of the band results from incomplete acid conversion;
- two strong bands around 1175 and 1115 cm^−1^ (**D** and **E**) of the C-O stretching vibrations of the ester.

From the included spectrum, no dehydration reaction leading to the formation of aconitic acid from citric acid is observed. Characteristic bands at a wavenumber of approximately 1600 cm^−1^ originating from the double C=C bond in the aconitic acid structure are absent. It can be further concluded that the addition of the PTSA catalyst does not noticeably accelerate the dehydration reaction.

**Figure 4:**
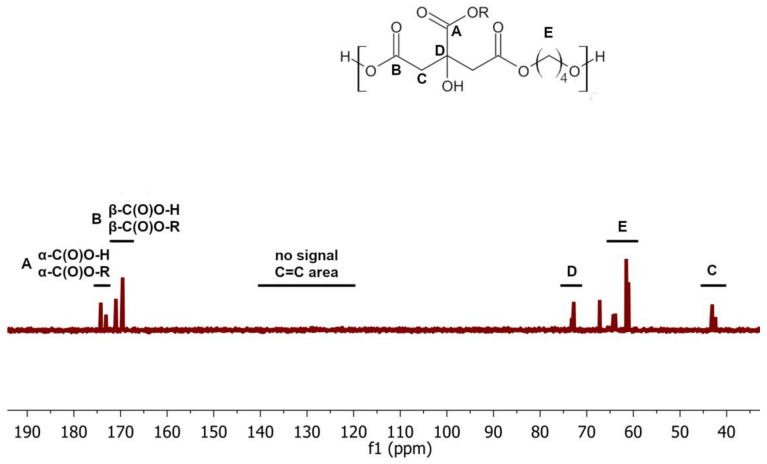
^13^C-NOE NMR spectrum of selected poly(1,4-butanediol citrate) sample.

Nonetheless, detecting a C=C bond band on the FTIR spectrum may be hampered by the presence of a stretching vibration band of the carbonyl group at similar wavenumber values. The absence of aconitic acid in the sample was further confirmed by the ^13^C NMR spectrum (Figure 4). No signal characteristic of the presence of double C=C bonds was observed on the spectrum.

Indeed, one should be aware that there is a high possibility of the formation of small, hardly detectable amounts of aconitic acid or other by-products. Additionally, given the colourless form of the obtained polyester samples, the presence of a large quantity of chromophore groups can be excluded; thus, the content of by-products can be considered to be negligibly small.

### 2.2. Mathematical Modelling of the Synthesis

The design of the experiment’s method was used to model the synthesis. A factorial plan 2^2^ was extended by centre and star points, resulting in a plan of 12 experiments. The experiments were carried out in random order to avoid systematic errors. Table 1 shows the area of the experiment, including the variables’ coding.

Calculations were performed using the StatSoft Statistica software (ver. 13.3.721.0). The functions were determined, describing the dependence of the degree of esterification of the acid groups (distinguishing α-C(O)O-H and β-C(O)O-H) on the process parameters: temperature (*x*_1_) and COOH/OH ratio (*x*_2_). The regression equations are presented for the input variables in coded form and include only statistically significant parameters (*p* = 0.05). *X_α_*, *X_β_* and *X_COOH_* denote the esterification degree of α-C(O)O-H, β-C(O)O-H and the overall COOH groups, respectively.

(1) $$X_{\alpha }=0.504+0.128x_{1}-0.067x_{2},$$

(2) $$X_{\beta }=0.679-0.144x_{2}-0.024x_{1}x_{2},$$

(3) $$X_{COOH}=0.623+0.028x_{1}-0.122x_{2}-0.026x_{1}x_{2},$$

**Figure 5:**
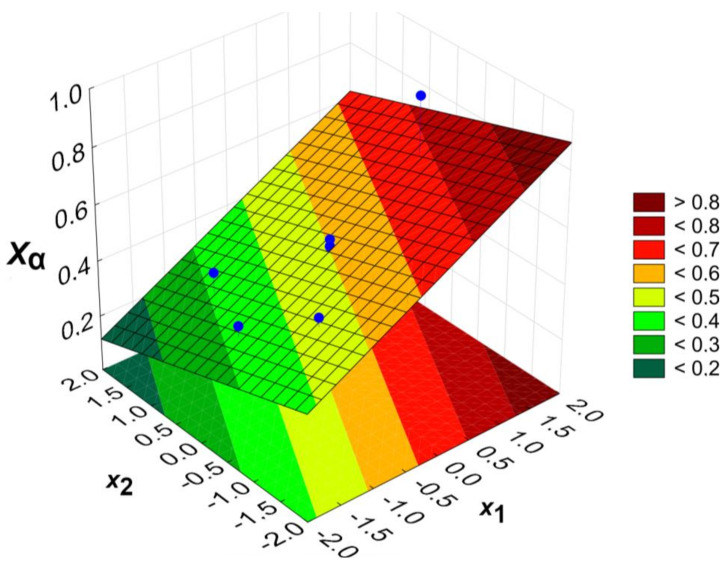
The dependence of the esterification degree of α-C(O)O-H groups (*X_α_*) on temperature (*x*_1_) and COOH/OH molar ratio (*x*_2_).

**Figure 6:**
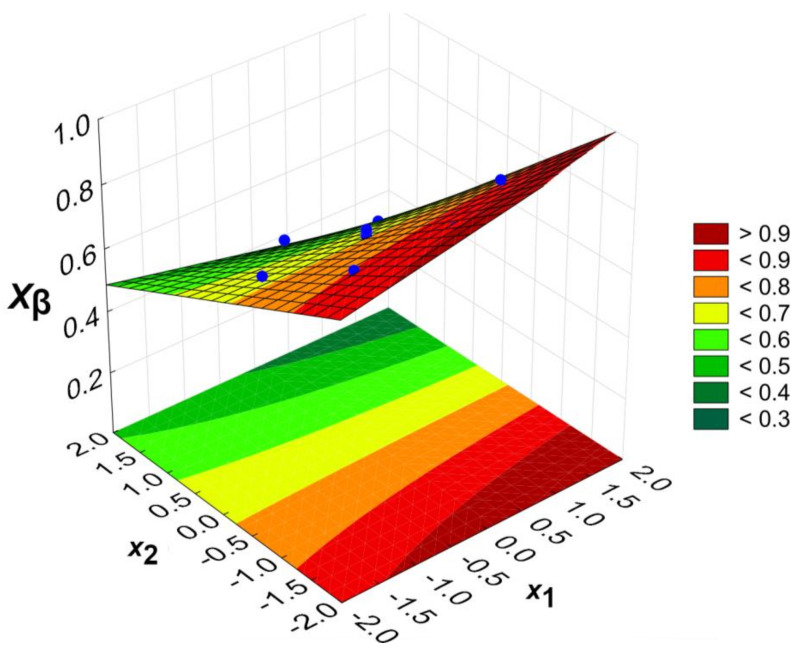
The dependence of the esterification degree of β-C(O)O-H groups (*X_β_*) on temperature (*x*_1_) and COOH/OH molar ratio (*x*_2_).

**Figure 7:**
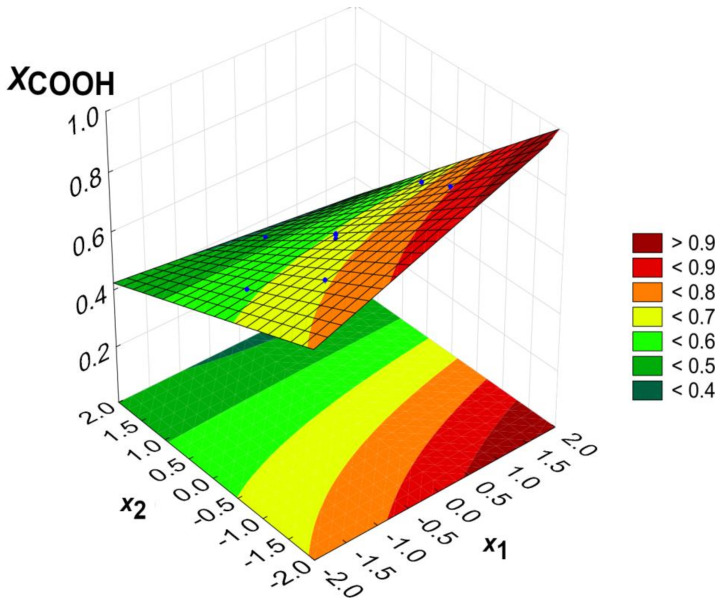
The dependence of overall esterification degree of acid groups (*X_COOH_*) on temperature (*x*_1_) and COOH/OH molar ratio (*x*_2_).

The calculated models are presented graphically as response surface plots (Figure 5, Figure 6 and Figure 7). Blue dots represent experimental data points. It is worth pointing out that the parameter space (temperature and the COOH/OH ratio) presented in Figure 5, Figure 6 and Figure 7 exceeds the experimentally measured values. The extrapolation may not always predict the correct results, and the model in this area is substantially less reliable than at the centre of the experimental plan. However, the optimum conditions were found within an area where experimental data exists.

The degree of esterification of α-C(O)O-H increases along with the increasing temperature and the growing excess of alcohol. For a *x*_1_ and *x*_2_ equal to 0, *X_α_* is about 50%. The value of the regression coefficient of *x*_1_ is almost twice that of the regression coefficient of *x*_2_, so a temperature change will have a greater effect on the change in *X_α_* values. The highest *X_α_* can be achieved for the combination of the highest temperature and lowest COOH/OH ratio due to an evident impact of the reaction kinetics (the collision theory). At the same time, an under-abundance of carboxyl groups favours the esterification of more sterically hindered and less reactive groups.

The esterification degree of the β-C(O)O-H groups is mainly influenced by the COOH/OH molar ratio (*x*_2_). The temperature (*x*_1_) has only a minor influence, as this parameter only occurs as part of the synergistic effect. This is because, already at lower temperatures, beta groups will be readily esterified as being more reactive than alpha groups. The lower reactivity of the α-C(O)O-H than the β-C(O)O-H groups is evidenced by the theoretical impossibility of obtaining their complete esterification in the described space of the input variables.

The model of the overall acid group’s esterification is essentially the outcome of the effects described for *X_α_* and *X_β_*. Both the excess hydroxyl groups and the high temperatures favour an easier esterification of the carboxyl groups of citric acid. However, it should be noted that esterification at 100% means the gelling of the reaction mixture, which should be avoided at the synthesis step.

A significant excess of hydroxyl groups will result in citric acid mono-, di- and tri-esters rather than a polymer or oligomer. Nonetheless, it seems doubtful that the described synthesis would yield chains with more than a dozen repeat units. The formation of oligomers is more likely, which only form a three-dimensional crosslinked structure upon gelling. Therefore, the optimal approach should be to use an equimolar COOH/OH ratio or a small excess of OH and choose a reaction temperature that limits the risk of gelling. Thus, the prepolymer can be subsequently modified, i.e., through thermal crosslinking.

On the basis of the experiments performed, the gelation of the reaction mixture should be expected at approximately *X_α_* = 70% and *X_β_* = 80%. According to this statement, the previously described mathematical models were used to calculate the optimal parameters of the synthesis, defined as being suitable for obtaining a product with a relatively high degree of esterification but that is not yet gelled. The parameters, in natural variables, were estimated as follows: temperature 141 °C and COOH/OH molar ratio equal to 0.6. Additional synthesis was carried out, and the product was characterised.

The product from the optimum point was a colourless resin of medium viscosity with the esterification degree of carboxyl groups equal to 0.80. Its estimated molecular weight is 1330 Da.

A good match between the calculated values and experimental results can be confirmed based on the results obtained. This ensures the applicability of the described synthesis models in further studies, allowing for a product with specific properties to be obtained.

### 2.3. Polymer Films Preparation and Study

Polymer from the optimum point (COOH/OH = 0.6) was used to prepare polymer films. Films with a 1.0 COOH/OH ratio have also been made for comparison.

The films were prepared via thermal crosslinking at a constant temperature of 115 °C. Different crosslinking time durations and their effect on the resulting material’s gel content and mass absorbability were investigated. The results are presented in Table 2.

It is apparent that higher crosslinked phase contents (as measured by the gel content parameter) can be obtained for films with excess hydroxyl groups under comparable conditions. This is most likely due to the higher flexibility of the structure and the lower network density in the prepolymer before thermal crosslinking. It is significant enough that, in the case of an equimolar COOH/OH ratio, 5 h of thermal crosslinking was too short to obtain a film.

The trend in the changes in the mass absorbability is not entirely clear, but is generally related to the content of the uncrosslinked phase. As the gel content increases, i.e., the amount of free hydrophilic end-chain groups decreases, the amount of polar liquid that can be bound within the structure decreases slightly. However, the amounts are still sufficient for it to be suggested that such materials could find use, for example, as carriers for biological fluids.

Given the differences in the gel content, only films crosslinked for at least 12 h were studied for their potential non-cytotoxicity. It was assumed that the rest would acidify the cell culture medium too rapidly.

**Figure 8:**
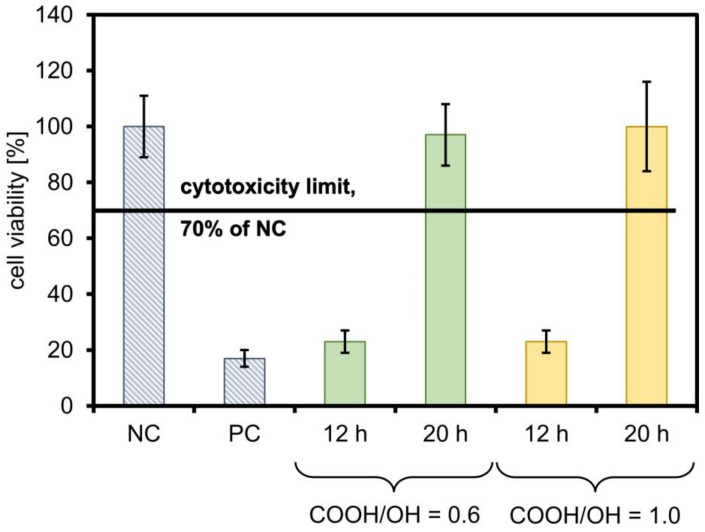
Cytotoxic effect of the 24-h extracts of the film as a percentage of the negative control (NC) viability. The black line shows the cytotoxicity limit at 70% of NC. Positive control (PC) is the result of the activity of the cells cultured with TritonX-100.

The cytotoxicity effect of the films was evaluated according to the results of the colourimetric XTT reagent assay using L929 mouse fibroblasts. The viability was determined based on the spectrophotometrically measured absorbance at 450 and 660 nm in the XTT test, following ISO EN 10993-5:2009. The results of the XTT cytotoxicity testing are presented in Figure 8.

The extracts were measured for their pH values, and the correlation is clear: the materials that were assessed as cytotoxic were slightly acidic (pH about 5.5), whereas the non-cytotoxic film extracts had a neutral pH. Films crosslinked for 20 h can be regarded as non-cytotoxic, regardless of the ratio of functional groups. Therefore, the gel content value alone is insufficient for assessing the potential applicability of such a film for cell culture. Despite the higher content of the crosslinked phase, the presence of a large amount of unreacted hydroxyl end-groups can have a negative effect. Although they do not acidify the culture medium, they make the network density in the film lower; therefore, the medium’s ease of penetration into the structure is more remarkable.

It Is possible that in the case of films containing COOH/OH = 1.0, despite their lower gel content, the leached soluble phase contains fractions with relatively long chains and a high degree of esterification of the acidic groups, which do not degrade fast enough to cause cell death. However, a cytotoxic effect may be noticeable with extended extraction and/or cell culture times. This is an interesting starting point for further research.

## 3. Conclusions

According to various studies, poly(citrate diols) show great potential as biomaterials in tissue engineering, especially for the regeneration of soft tissues such as blood vessels. The most widely described is poly(1,8-octanediol citrate). However, its limitation is hydrophobicity due to the long carbon chain in the diol molecule, which can reduce cell adhesion to the material. In order to increase the hydrophilicity of the polymer, it was decided to replace 1,8-octanediol with another aliphatic dihydroxy alcohol with a shorter chain—namely, 1,4-butanediol.

The synthesis of poly(1,4-butanediol citrate) in the presence of the PTSA catalyst was carried out, and the successful esterification was confirmed through spectral analysis. Based on the obtained data, the occurrence of the gel point of the reaction mixture was estimated. It was shown that the catalyst addition used did not noticeably contribute to the occurrence of undesired competing reactions.

The effects of the temperature and COOH/OH functional group ratio on the selected product properties were investigated. Mathematical synthesis models were calculated, and the optimum conditions were determined. A good fit between the model and the experimental results was confirmed. The products of the described reaction are oligomers with several repeating units in the chain.

Polymer films were obtained from selected synthesis products through thermal crosslinking. It was shown that the ratio of functional groups significantly affects the insoluble phase (gel) content of the resulting film. This is subsequently of great importance for studies using cells, which are highly sensitive to any change in the ambient pH. The most important characteristics of this type of material include: (1) the content of the crosslinked phase; (2) the presence of free acidic end groups; and (3) the density of the crosslinking and—associated with it— the rate and ease of penetration of the medium that hydrolyses the bonds in the polymer chain. These findings can be supported by the existing literature on similar biomaterials, regarding, e.g., β-keratin, showing the impact of the number of functional groups on the solubility, mechanical behaviour and the ability to form a three-dimensional network, which corresponds to the gelation of the polyester [34].

## 4. Materials and Methods

1,4-butanediol (Thermo Scientific, 99%, Bremen, Germany), anhydrous citric acid (Acros Organics, ≥99.5%, Swindon, UK) and *p*-toluenesulfonic acid (PTSA; Sigma Aldrich, ≥98.5%, Lisbon, Poland) monohydrate were used without prior preparation.

### 4.1. Synthesis Procedure

Poly(1,4-butanediol citrate) was synthesised in a Mettler Toledo MultiMax parallel reactors system. Hastelloy reactors with a working capacity of 50 mL with a Teflon cover equipped with a mechanical stirrer, temperature sensor and DeanStark apparatus were used. The reaction mixture was heated for 10 min to the set temperature, which was then held constant for 40 min. After the reaction, the product was allowed to cool down to room temperature.

### 4.2. FTIR

The IR/ATR spectra were recorded on a Bruker FTIR ALPHA II spectrometer. Overall, 32 scans in the range of 400–4000 cm^−1^ were performed for each sample.

### 4.3. NMR

The spectra were recorded on an Agilent 400 MHz NMR spectrometer. The samples were prepared by dissolving approx. 150 mg of the sample in 1 mL of deuterated acetone for 24 h.

Analysis of the ^13^C-NOE NMR spectra was used to calculate the degree of conversion of the carboxyl groups of citric acid, according to the following formulas:

(4) $$X_{\alpha }=\frac{\int E_{\alpha }}{\int E_{\alpha }+\int A_{\alpha }}\cdot 100\%,$$

(5) $$X_{\beta }=\frac{\int E_{\beta }}{\int E_{\beta }+\int A_{\beta }}\cdot 100\%,$$

(6) $$X_{COOH}=\frac{\int E_{\alpha }+\int E_{\beta }}{\int E_{\alpha }+\int A_{\alpha }+\int E_{\beta }+\int A_{\beta }}\cdot 100\%,$$

where: *X_α_*—conversion of α-C(O)O-H groups; *X_β_*—conversion β-C(O)O-H groups; *X_COOH_*—total conversion of acid groups; ∫ *E_α_* or ∫ *E_β_*—integral value of the signal of the ester α-C(O)O-R or β-C(O)O-R groups; ∫ *A_α_* or ∫ *A_β_*—integral value of the signal of the acid α-C(O)O-H or β-C(O)O-H groups.

### 4.4. Polymer Film Preparation

The prepolymer mass was poured into a flat, rectangular Teflon mould. The mould with the prepolymer was placed in a laboratory dryer at 115 °C for 5, 8, 12 or 20 h.

### 4.5. Gel Content and Mass Absorbability

Circular pieces with a diameter of 1 cm were cut from polymer films. The specimens were weighed, placed in Falcon-type plastic tubes and then flooded with 5 mL of PBS buffer. The tubes were placed for 24 h on a Heidolph reciprocating motion shaker equipped with a thermostatic chamber. During the test, a constant temperature of 25 °C and a rotation speed of 150 rpm were maintained. Wet samples were weighed immediately after removal from the test tube, then dried and weighed again. Drying at room temperature under 20 mbar for 96 h was carried out. The gel content, i.e., the crosslinked (gelled) polymer content, was determined:

(7) $$gel\;content=\frac{dried\;sample\;weight}{initial\;sample\;weight}\times 100\%,$$

It was also determined how much liquid the material could hold within:

(8) $$mass\;absorbability=\frac{wet\;sample\;weight}{dried\;sample\;weight}\times 100\%,$$

The results from the three trials were averaged.

### 4.6. Cytotoxicity

Cytotoxicity tests were performed using mouse fibroblast line L929 (ATCC). All procedures were carried out in a UV-sterilised laminar flow cabinet and using 1 g/L Dulbecco’s Modified Eagle Medium (DMEM) medium, supplemented with 10% *v*/*v* inactivated fetal bovine serum and 1% *v*/*v* Pen-Strep. All culture media were supplied by Gibco.

#### 4.6.1. Maintaining L929 Cells

The culture medium used was aseptically pipetted out from culture flasks containing L929 cells. The remaining cell monolayer was rinsed twice with sterile Dulbecco’s Phosphate Buffered Saline (DPBS) without Ca^2+^ and Mg^2+^. Then, 3 mL of sterile 0.05% trypsin-EDTA was poured into each flask. The flasks were subsequently incubated for 5 min at 37 °C. The suspension of the detached cells was transferred to a sterile Falcon-type centrifuge tube, 10 mL of fresh culture medium was added and the tube was centrifuged at 4500 min^−1^ for 5 min. The supernatant was aseptically removed, and the cells were suspended in a 10 mL fresh culture medium. Cells were counted in a hemocytometer, passaged into new culture flasks and continuously incubated at 37 °C in a 5% CO_2_-enriched air atmosphere.

#### 4.6.2. XTT Cytotoxicity Test

Discs of 1.7 cm diameter were cut from the films and placed in 24-well plates. The plates were vacuum-packed and sterilised. Next, 1.8 mL of culture medium was poured into each well. The plates were incubated at 37 °C for 24 h.

A cell suspension of 10^5^ cells/mL was placed in a 96-well plate, 100 μL per well. The outer wells were flooded with DPBS to prevent evaporation of the culture medium. The plates were incubated at 37 °C for 24 h. The medium was removed, and the extracts were added, 100 μL per well. Some wells were flooded with fresh medium (negative control) or 0.1% solution of the Triton X-100 in DMEM (positive control). The plate was subsequently incubated at 37 °C for 24 h in a 5% CO_2_-enriched air atmosphere.

After the incubation, the culture medium used was removed, and the cells were washed twice with 100 μL of DPBS. XTT reagent (CyQUANT XTT Cell Viability Assay, ThermoFisher Scientific) solution in culture medium (33% *v*/*v*) was added, 150 μL per well. The 96-well plate was placed in an incubator (37 °C) for 4 h. The absorbance was automatically measured in the multi-well plate reader for 450 and 630 nm (reference wavelength). The results were averaged and compared to the negative control, which was assumed as 100% of the metabolic activity of the cells.

## Figures and Tables

**Table 1:** Values of the input variables in the experiment area.

Variable	−1.41	−1.00	0.00	+1.00	+1.41
temperature [°C]	116	120	130	140	144
COOH/OH ratio [mol/mol]	0.3	0.5	1.0	1.5	1.7

**Table 2:** Gel content and mass absorbability of polymer films with different COOH/OH molar ratios.

		Crosslinking Time [h]	5	8	12	20
COOH/OH ratio	0.6	Gel content [%]	96 ± 1	96 ± 2	98 ± 2	98 ± 1
		Mass absorbability [%]	159 ± 7	129 ± 9	139 ± 14	123 ± 2
	1.0	Gel content [%]	not sufficiently crosslinked	86 ± 2	87 ± 1	90 ± 1
		Mass absorbability [%]		139 ± 5	145 ± 5	141 ± 5

## Data Availability

The data presented in this study are available on request from the corresponding author.
